# Supplementary material for: Effects of L-valine in layer diets containing 0.72% isoleucine
Source: PLoS One. 2022 Apr 13;17(4):e0258250. doi: 10.1371/journal.pone.0258250 (PMC9007362; doi:10.1371/journal.pone.0258250)
Supplement: Attachment — Submitted filename: Valin review and comments 8 18 21.docx [file pone.0258250.s001.docx]

-------------------------------

Changes to Manuscript

Introduction
1- Need more information about the effects of isoleucine in layer hens

**Pages 1 -3**

2- recently references for using Valine in layer diets

**P 4, line 9 - 19**

**P 5, lines 4 - 11**

Discussion
 In this part you focused on the studies which agree or disagree with your results without any explanation why these findings were agree with disagree with you.

**Please see improved Results and discussion, P 5 - 13,**

4- You must write the mode of action of L-valine and isoleucine on layer performance and egg quality

**P 11, lines 9 - 18**

5- I advise to use some recently reference for using amino acids in poultry and the additives affect the egg quality and immunity
I recommend reading and using the following references:
Saleh A. A. (2016) Effect of Low-Protein in Iso-Energetic Diets on Performance, Carcass Characteristics, Digestibilities and Plasma Lipids of Broiler Chickens. Egyptian Poultry Science Journal. Vol (36) (I): (251-262).
Saleh A. A, Mohammed S. Eltantawy , Esraa M. Gawish , Hassan H. Younis , Khairy A. Amber , Abd El-Moneim E. Abd El-Moneim & Tarek A. Ebeid (2020) Impact of Dietary Organic Mineral Supplementation on Reproductive Performance, Egg Quality Characteristics, Lipid Oxidation, Ovarian Follicular Development, and Immune Response in Laying Hens Under High Ambient Temperature. Biological Trace Element Research. 195:506–514.
Saleh, AA., Ashia Zaki, Ahmed El- Awady, Khairy Amber, Neamat Badwi, Yahya Eid, Tarek A. Ebeid (2020) Effect of substituting wheat bran with cumin seed meal on laying performance, egg quality characteristics and fatty acid profile in laying hens. Veterinarski Arhiv 90 (1), 47-56,
Saleh A. A., Abeer A. Kirrella, Mahmoud A. O. Dawood, Tarek A. Ebeid (2019) Effect of dietary inclusion of cumin seed oil on the performance, egg quality, immune response and ovarian development in laying hens under high ambient temperature. Animal Physiology and Animal Nutrition. 103(6):1810-1817.

**Please see several references in the Introduction and Results and Discussion.**

6- Material and Methods

Why didn’t you prepare your diets according the layer strain catalog (LSL-LITE)?

**Diets were prepared using the least expensive and available protein sources. See p 15, Table 5.**

P13 L1 Mash and fresh water were provided ad libitum, what is mean of mash are you mean feed form?

**P 15, line 3.**

Table 5. Feed and nutrient composition of diets.
You must provide the crude protein levels of (Canola Meal, Sunflower seed meal, Soybean Meal and Poultry By-Product Meal

**P 14, new Table 5.**

**----------------------------------**

Reviewer #2: This is the review for the manuscript PONE-D-20-35025, entitled “L-valine in diets with 0.72% isoleucine: effect on production, egg quality, serum biochemistry, and ileal digestibility of protein” for PLOS ONE Journal.
The manuscript aims at investigating the effect of dietary supplementation of L-valine on laying hen performance in terms of egg production and quality, serum biochemistry, and protein ileal digestibility.
I have the following more detailed comments that can help authors in the revision of the manuscript:
Page 4 (Table 1): Kindly check body weight values for laying hens. It seems not correct. These values more appropriate for egg weight not hen body weight.

**See change in Table 1.**

P 8 (Line 5): delete this sentence.

**Deleted**

Page 10 (Line 16): The effect of valine: lysine on egg production is not discussed. The authors only compared with the findings in the previous studies and nothing mentioned why this alteration has occurred.

**See a more extensive discussion for Results and discussion and on p 11, line 9 to 18.**

Page 12 (Material and methods)
Page 12 (Line 19): 490 refers to what? Kindly write (N=490). Kindly provide the average initial hen weight.

**P 14, line 18**

Nothing mentioned about blood or serum collection or analysis, and ileal digestibility, egg quality assessment. **Information is in the companion paper.**

Kindly check the superscripts according to the pooled SE and presented values of mean through the whole manuscript. **Please see Results and discussion.**
